# Editorial to the Special Issue “The Effect of Exercise Intensity and Psychological Intervention on Musculoskeletal Disorders”

**DOI:** 10.3390/ijerph20105777

**Published:** 2023-05-10

**Authors:** Eleuterio A. Sánchez-Romero, Miguel de-Pedro, Josué Fernández-Carnero

**Affiliations:** 1 Department of Physiotherapy, Faculty of Sport Sciences, Universidad Europea de Madrid, 28670 Villaviciosa de Odón, Spain; 2 Physiotherapy and Orofacial Pain Working Group, Sociedad Española de Disfunción Craneomandibular y Dolor Orofacial (SEDCYDO), 28009 Madrid, Spain; 3 Musculoskeletal Pain and Motor Control Research Group, Faculty of Sport Sciences, Universidad Europea de Madrid, 28670 Madrid, Spain; josue.fernandez@urjc.es; 4 Department of Physiotherapy, Faculty of Health Sciences, Universidad Europea de Canarias, 38300 Santa Cruz de Tenerife, Spain; 5 Musculoskeletal Pain and Motor Control Research Group, Faculty of Health Sciences, Universidad Europea de Canarias, C/Inocencio García 1, 38300 La Orotava, Spain; 6 Department of Clinical Dentistry, Faculty of Biomedical Sciences, Universidad Europea de Madrid, 28670 Madrid, Spain; miguel.depedro@universidadeuropea.es; 7 Department of Physical Therapy, Occupational Therapy, Rehabilitation and Physical Medicine, Universidad Rey Juan Carlos, 28922 Alcorcón, Spain; 8 Motion in Brains Research Group, Institute of Neuroscience and Sciences of the Movement (INCIMOV), Centro Superior de Estudios Universitarios La Salle, Universidad Autónoma de Madrid, 28023 Madrid, Spain

## 1. Introduction

Musculoskeletal disorders are responsible for the most prevalent form of pain, and necessitate a comprehensive approach to rehabilitation [1]. From a therapeutic standpoint, they can vary from myofascial pain syndrome that produces or adds to craniofacial, low back, shoulder, and knee pain, to intervertebral joints that cause or contribute to comparable discomfort and cervical pain.

Moreover, peripheral nervous tissue is frequently impacted by locomotor system injuries or accumulated overloads, both in sports activities and in the execution of professional or daily routines.

All of these complaints, and many others connected to them, call for intervention from various disciplines, such as physiotherapy, dentistry, and psychology.

This Special Issue contains five articles that address various issues related to musculoskeletal disorders, pain management, and rehabilitation. Among these articles, two original research focus on temporomandibular disorders (TMD) and psychological factors, while another examines post-COVID-19 patients from the first and second waves and their treatment with multicomponent exercise, with a follow-up two years. Furthermore, a study protocol with preliminary results explored the management of fibromyalgia patients with manual therapy, and a systematic review and meta-analysis investigated the use of a placebo in manual therapy.

Overall, these studies highlight the importance of improving the quality of life for patients with musculoskeletal disorders by expanding the existing evidence base. The findings also provide valuable insights for future research, pointing towards areas where more investigation is needed. By addressing these issues, healthcare professionals can provide more comprehensive and effective treatments to help patients manage their pain and improve their overall well-being.

## 2. Musculoskeletal Disorders in the Craniomandibular Region

The dentist plays an important role in the interdisciplinary management of musculoskeletal disorders in the orofacial region. Dental therapy is varied, as there are a number of musculoskeletal conditions that affect the temporomandibular joint and the masticatory muscles [2]. New muscular and intraarticular infiltration techniques are being added to the classic use of occlusal splints, without forgetting the acute pharmacological management of emergencies in consultation [3] and the self-care advice that all components of the therapeutic team must transmit [4]. The effectiveness of occlusal splints has been debated in recent years [5], demonstrating their effectiveness in improving mandibular range movement and decreasing muscular pain [6,7]. The use of these splints should never be established as a unimodal treatment. It should be recommended together with the management of the pathology by the physiotherapist. On certain occasions, the infiltration of anesthetics by the dentist has managed to improve myofascial pain in the masticatory muscles [8], as well as the use of botulinum toxin, which can reduce symptoms related to muscular motor activity [9]. Hyaluronic acid infiltrations, accompanied by mandibular mobilization exercises, have proven to be very effective for intra-articular hypomobility disorders such as intermittent locks and degenerative alterations [10].

TMD and migraines, though poorly understood, are often associated with of various elements such as common physiology, genetics, and environmental influences. TMD encompasses clinical issues related to the musculature used in chewing, the temporomandibular joint, and related structures. Migraine is a chronic neurological disorder characterized by recurrent headaches and associated symptoms. TMD and headaches are strongly correlated, with approximately 67% of individuals with TMD experiencing headaches. Although primary headaches, such as migraines, are more prevalent in individuals with TMD symptoms, those who suffer from both headaches and TMD tend to experience significantly higher levels of pain and disability compared to those with TMD alone. Psychological factors, such as anxiety, depression, and maladaptive coping, are common in both pathologies. The triad of psychological factors, TMD myalgia and migraine, requires further study to understand the relationships between the three, emphasizing the differentiation of the groups studied. Overall, the main goal of Viñals Narváez et al. [11] was to describe the psychological and behavioral factors involved in TMD myalgia and migraine.

The consensus on bruxism characterizes it as an activity of the masticatory muscles, regulated by the central nervous system, with peripheral factors playing a secondary role. There are two types of bruxism: awake bruxism and sleep bruxism. Anxiety, stress, and depression have been associated with bruxism. While not typically considered pathological, bruxism (teeth grinding) can potentially result in issues such as tooth damage and orofacial pain in certain situations. It is also considered as one of the possible risk factors in the development of TMD. Recent evidence suggests that the COVID-19 pandemic has intensified bruxism and TMD symptoms, probably due to psychological and emotional stress. The aim of Osses-Anguita et al. [12] was to examine the effects of the COVID-19 pandemic on potential sleep and awake bruxism, as well as the associated psychological factors, among first-year dentistry students, using validated and reliable questionnaires. It was hypothesized that higher levels of possible awake and sleep bruxism would be observed in the group affected by the pandemic (2020/21), as compared to the pre-pandemic (2018/19) and post-pandemic groups (2021/22).

## 3. Multicomponent Exercise Program in Recovering from the Immediate Sequelae of COVID-19

The COVID-19 pandemic was declared by the World Health Organization (WHO) in March 2020 due to its highly contagious nature and severe impact [13]. The pandemic has affected millions of people, with over 300 million cases and more than 5 million deaths reported so far. While most people experience mild symptoms, a significant proportion suffer from multisystemic symptoms that require intensive care and a multidisciplinary approach. Diagnosis of COVID-19 can be aided by three diagnostic tools: the PCR, antigen, and antibody tests. These tests are crucial for detecting and confirming active infections and for ensuring appropriate treatment and sanitation protocols [14]. Symptoms of COVID-19 include fever, headache, musculoskeletal pain, cough, pneumonia, dyspnea, and digestive issues [15]. The severity and duration of these symptoms depend on various factors, including the patient’s health status and comorbidities. The pandemic has led to unprecedented changes in healthcare, with healthcare professionals facing new challenges in treating and rehabilitating COVID-19 patients [16]. Multicomponent exercise programs (MEPs) have emerged as effective interventions for improving effort tolerance and reducing post-COVID-19 sequelae [17]. The aim Martínez Rolando et al. [18] was to evaluate the impact of a Multidisciplinary Exercise Program (MEP) on cardiorespiratory performance, health status, disability resulting from dyspnea, aerobic capacity, endurance, and immediate sequelae of COVID-19. MEPs have the potential to provide an economical and effective therapy for post-COVID-19 neuromusculoskeletal sequelae, particularly in vulnerable populations.

## 4. Manual Therapy and Musculoskeletal Pain

Fibromyalgia (FM) is a persistent syndrome characterized by widespread musculoskeletal pain, fatigue, as well as sleep and mood, affecting 2.1% of the global population, with a higher prevalence in women. The costs associated with FM are high, including direct medical costs as well as indirect costs such as decreased work hours, sick leave, and permanent disability. Although the pathogenesis of FM is not fully understood, various factors such as nervous system dysfunctions, immune system abnormalities, endocrine system disturbances, environmental stressors, and psychiatric or psychological issues have been proposed as potential factors.

Pharmacological treatments are usually ineffective, and pain-modulating drugs, exercise, aquatic exercise, manual therapy, needle therapies, and patient education are commonly used. Manual therapy, including myofascial techniques and accessory joint mobilizations, has shown promising results in improving pain, sleep, and quality of life compared to sham in patients with FM. The aim of Romane Audoux et al. [19] was to determine which manual therapy approach is more effective in improving symptoms in patients with fibromyalgia.

Pain is a significant burden for millions of Europeans and can lead to depression. Manual therapy, such as joint mobilization and neural mobilization, is commonly used to manage musculoskeletal pain, but some believe that the positive effects are due to the placebo effect rather than the therapy itself. Placebo-controlled randomized clinical trials (RCTs) incorporate a sham control group to replicate the treatment under investigation, providing a reliable comparator. The objective of Molina-Álvarez et al. [20] was to assess whether the design of placebo control groups can lead to divergent interpretations of the effectiveness of manual therapy techniques. The sham group must be non-active and harmless, ensuring the blinding of patients and control of biases, among other methodological factors, to determine treatment effects accurately. The review also sought to determine if the simulated groups utilized in the literature were designed to yield dependable comparisons.

## 5. Conclusions

This Special Issue offers a wealth of information on some of the most significant and widespread musculoskeletal disorders, providing readers with the opportunity to expand their understanding and knowledge of their intricacies. The articles presented within this issue provide a comprehensive view of these disorders and their effects, allowing readers to gain insights into their complexity and the various approaches taken to address them.

## Data Availability

Not applicable.
